# Supplementary material for: Multi‐dataset identification of innovative feature genes and molecular mechanisms in keratoconus
Source: J Cell Mol Med. 2024 Sep 19;28(18):e70079. doi: 10.1111/jcmm.70079 (PMC11412914; doi:10.1111/jcmm.70079)
Supplement: Supplementary file 2 — Material S2. [file JCMM-28-e70079-s002.zip › 7.GSEA analysis/2.ATOH7/KEGG_PARKINSONS_DISEASE.html]

Details for gene set KEGG\_PARKINSONS\_DISEASE[GSEA]

|  || Dataset | ATOH7.group.cls#h\_versus\_l |
| Phenotype | group.cls#h\_versus\_l |
| Upregulated in class | h |
| GeneSet | KEGG\_PARKINSONS\_DISEASE |
| Enrichment Score (ES) | 0.39711964 |
| Normalized Enrichment Score (NES) | 1.6808902 |
| Nominal p-value | 0.026266417 |
| FDR q-value | 0.27788216 |
| FWER p-Value | 0.424 |
Table: GSEA Results Summary

  

Fig 1: Enrichment plot: KEGG\_PARKINSONS\_DISEASE      
 Profile of the Running ES Score & Positions of GeneSet Members on the Rank Ordered List

  

| SYMBOL | TITLE | RANK IN GENE LIST | RANK METRIC SCORE | RUNNING ES | CORE ENRICHMENT || 1 | SNCAIP | na | 46 | 0.870 | 0.1504 | Yes |
| 2 | SNCA | na | 94 | 0.777 | 0.2846 | Yes |
| 3 | LRRK2 | na | 1108 | 0.431 | 0.3304 | Yes |
| 4 | COX8C | na | 1278 | 0.411 | 0.3971 | Yes |
| 5 | SLC18A1 | na | 3546 | 0.254 | 0.3759 | No |
| 6 | APAF1 | na | 7669 | 0.126 | 0.2788 | No |
| 7 | NDUFA4L2 | na | 8390 | 0.110 | 0.2773 | No |
| 8 | SLC25A4 | na | 9319 | 0.093 | 0.2668 | No |
| 9 | SLC25A31 | na | 9626 | 0.088 | 0.2732 | No |
| 10 | HTRA2 | na | 10364 | 0.076 | 0.2651 | No |
| 11 | VDAC1 | na | 11345 | 0.061 | 0.2475 | No |
| 12 | CYC1 | na | 11419 | 0.060 | 0.2558 | No |
| 13 | UBE2G1 | na | 11483 | 0.059 | 0.2643 | No |
| 14 | SDHB | na | 11751 | 0.056 | 0.2664 | No |
| 15 | UBA1 | na | 11770 | 0.055 | 0.2755 | No |
| 16 | SLC6A3 | na | 12195 | 0.050 | 0.2720 | No |
| 17 | PARK7 | na | 12292 | 0.049 | 0.2778 | No |
| 18 | UBB | na | 12445 | 0.047 | 0.2815 | No |
| 19 | UBE2L6 | na | 12582 | 0.045 | 0.2854 | No |
| 20 | COX7B2 | na | 12668 | 0.044 | 0.2907 | No |
| 21 | UQCRB | na | 13482 | 0.035 | 0.2734 | No |
| 22 | NDUFS2 | na | 13540 | 0.035 | 0.2778 | No |
| 23 | NDUFA6 | na | 13652 | 0.034 | 0.2805 | No |
| 24 | SLC25A6 | na | 13704 | 0.033 | 0.2849 | No |
| 25 | UQCRH | na | 13770 | 0.033 | 0.2887 | No |
| 26 | COX7A1 | na | 14036 | 0.030 | 0.2863 | No |
| 27 | CASP9 | na | 14196 | 0.029 | 0.2867 | No |
| 28 | NDUFV2 | na | 14197 | 0.029 | 0.2917 | No |
| 29 | COX4I1 | na | 14252 | 0.028 | 0.2950 | No |
| 30 | NDUFA2 | na | 14444 | 0.026 | 0.2941 | No |
| 31 | NDUFA4 | na | 14988 | 0.021 | 0.2821 | No |
| 32 | UQCRFS1 | na | 15042 | 0.021 | 0.2842 | No |
| 33 | COX6A1 | na | 15045 | 0.021 | 0.2877 | No |
| 34 | NDUFA7 | na | 15138 | 0.020 | 0.2886 | No |
| 35 | TH | na | 15211 | 0.019 | 0.2898 | No |
| 36 | COX7A2L | na | 15220 | 0.019 | 0.2930 | No |
| 37 | NDUFB7 | na | 15235 | 0.019 | 0.2959 | No |
| 38 | NDUFA3 | na | 15254 | 0.019 | 0.2987 | No |
| 39 | COX4I2 | na | 15306 | 0.018 | 0.3004 | No |
| 40 | SDHA | na | 15471 | 0.017 | 0.2987 | No |
| 41 | PPID | na | 15617 | 0.016 | 0.2972 | No |
| 42 | NDUFS7 | na | 15694 | 0.015 | 0.2977 | No |
| 43 | UBE2J2 | na | 15761 | 0.015 | 0.2983 | No |
| 44 | SDHD | na | 15796 | 0.014 | 0.2999 | No |
| 45 | COX5A | na | 15943 | 0.013 | 0.2979 | No |
| 46 | UQCR10 | na | 16088 | 0.012 | 0.2959 | No |
| 47 | UBE2G2 | na | 16193 | 0.011 | 0.2949 | No |
| 48 | UQCRC2 | na | 16211 | 0.011 | 0.2964 | No |
| 49 | CYCS | na | 16260 | 0.011 | 0.2968 | No |
| 50 | NDUFA9 | na | 16374 | 0.010 | 0.2953 | No |
| 51 | COX7C | na | 16469 | 0.009 | 0.2941 | No |
| 52 | NDUFB4 | na | 16810 | 0.006 | 0.2854 | No |
| 53 | UQCRQ | na | 16876 | 0.006 | 0.2845 | No |
| 54 | VDAC2 | na | 16978 | 0.005 | 0.2824 | No |
| 55 | NDUFB2 | na | 17205 | 0.003 | 0.2763 | No |
| 56 | COX8A | na | 17213 | 0.003 | 0.2766 | No |
| 57 | VDAC3 | na | 17448 | 0.001 | 0.2699 | No |
| 58 | NDUFB6 | na | 17476 | 0.000 | 0.2692 | No |
| 59 | NDUFB3 | na | 17733 | -0.002 | 0.2621 | No |
| 60 | NDUFB9 | na | 17840 | -0.003 | 0.2595 | No |
| 61 | NDUFA5 | na | 17888 | -0.003 | 0.2587 | No |
| 62 | NDUFC1 | na | 17903 | -0.003 | 0.2588 | No |
| 63 | COX6C | na | 17968 | -0.004 | 0.2576 | No |
| 64 | NDUFS8 | na | 18032 | -0.004 | 0.2566 | No |
| 65 | COX7A2 | na | 18090 | -0.005 | 0.2558 | No |
| 66 | SLC25A5 | na | 18293 | -0.007 | 0.2511 | No |
| 67 | NDUFB10 | na | 18344 | -0.007 | 0.2508 | No |
| 68 | UQCRC1 | na | 18411 | -0.008 | 0.2503 | No |
| 69 | NDUFA8 | na | 18467 | -0.008 | 0.2501 | No |
| 70 | NDUFS3 | na | 18481 | -0.008 | 0.2512 | No |
| 71 | COX5B | na | 18689 | -0.010 | 0.2470 | No |
| 72 | UBE2J1 | na | 18754 | -0.011 | 0.2470 | No |
| 73 | NDUFB8 | na | 18767 | -0.011 | 0.2485 | No |
| 74 | NDUFS4 | na | 18768 | -0.011 | 0.2504 | No |
| 75 | COX6B1 | na | 19017 | -0.013 | 0.2455 | No |
| 76 | NDUFAB1 | na | 19284 | -0.015 | 0.2405 | No |
| 77 | SDHC | na | 19318 | -0.016 | 0.2423 | No |
| 78 | COX7B | na | 19335 | -0.016 | 0.2446 | No |
| 79 | UQCR11 | na | 19468 | -0.017 | 0.2437 | No |
| 80 | NDUFV3 | na | 19559 | -0.018 | 0.2442 | No |
| 81 | NDUFS5 | na | 19842 | -0.020 | 0.2396 | No |
| 82 | NDUFA1 | na | 19847 | -0.020 | 0.2430 | No |
| 83 | NDUFV1 | na | 19894 | -0.021 | 0.2453 | No |
| 84 | NDUFC2 | na | 19991 | -0.022 | 0.2463 | No |
| 85 | NDUFB1 | na | 20064 | -0.022 | 0.2481 | No |
| 86 | UBA7 | na | 20242 | -0.024 | 0.2472 | No |
| 87 | NDUFS6 | na | 21061 | -0.032 | 0.2292 | No |
| 88 | PINK1 | na | 21213 | -0.034 | 0.2308 | No |
| 89 | UBE2L3 | na | 21237 | -0.034 | 0.2361 | No |
| 90 | NDUFB5 | na | 22347 | -0.047 | 0.2122 | No |
| 91 | NDUFS1 | na | 22850 | -0.053 | 0.2069 | No |
| 92 | UCHL1 | na | 22979 | -0.054 | 0.2127 | No |
| 93 | NDUFA10 | na | 23223 | -0.058 | 0.2158 | No |
| 94 | COX6B2 | na | 24380 | -0.075 | 0.1955 | No |
| 95 | CASP3 | na | 24828 | -0.083 | 0.1970 | No |
| 96 | GPR37 | na | 26510 | -0.115 | 0.1685 | No |
| 97 | SLC18A2 | na | 28219 | -0.157 | 0.1466 | No |
| 98 | COX6A2 | na | 30724 | -0.237 | 0.1156 | No |
Table: GSEA details [plain text format]

  

Fig 2: KEGG\_PARKINSONS\_DISEASE      
 Blue-Pink O' Gram in the Space of the Analyzed GeneSet

  

Fig 3: KEGG\_PARKINSONS\_DISEASE: Random ES distribution      
 Gene set null distribution of ES for **KEGG\_PARKINSONS\_DISEASE**
